# Supplementary material for: Interaction of Substrates with γ-Secretase at the Level of Individual Transmembrane Helices—A Methodological Approach
Source: Int J Mol Sci. 2023 Sep 21;24(18):14396. doi: 10.3390/ijms241814396 (PMC10531681; doi:10.3390/ijms241814396)
Supplement: Supplementary file 1 [file ijms-24-14396-s001.zip › ijms-2554147-supplementary.pdf]

Supplemental Information

Interaction of Substrates with γ-Secretase at the Level of Individual Transmembrane Helices—A Methodological Approach

Theresa M. Pauli †,‡ , Ayse Julius †, Francesco Costa § , Sabine Eschrig, Judith Moosmüller k , Lea Fischer , Christoph Schanzenbach ¶ , Fabian C. Schmidt, Martin Ortner * and Dieter Langosch *

Lehrstuhl für Chemie der Biopolymere, Technische Universität München, Weihenstephaner Berg 3, 85354 Freising, Germany; theresa.pauli@tum.de (T.M.P.); ayse.julius87@gmail.com (A.J.); fcosta@ebi.ac.uk (F.C.); sabine.eschrig@tum.de (S.E.); moosmueller.judith@gmx.de (J.M.); lea.fischer@tum.de (L.F.); christoph.schanzenbach@gmx.de (C.S.); fabian.c.schmidt@tum.de (F.C.S.)
* Correspondence: martin.ortner@tum.de (M.O.); langosch@tum.de (D.L.)
† These authors contributed equally to this work.
‡ Current address: Chair of Livestock Biotechnology, Technische Universität München, Liesel-Beckmann-Str. 1, 85354 Freising, Germany.
§ Current address: EMBL-EBI, Hinxton CB10 1SD, UK.
k Current address: Institut für Virologie, Trogerstr 30, 81675 München, Germany.
¶ Current address: Biotype GmbH, 01109 Dresden, Germany.

Table S1. Sequences and frames of all TMDs used in manual testting (approach 1).

**APP (A4_human)**	NMR[1]	...SNK**GAIIGLMVGGVVIATVIVITLVMLKK**KQY...[1]
	frame[2] 0	...GAIIGLMVGGVVIATVIVITLVML
	frame 1	..LGAIIGLMVGGVVIATVIVITLVM.
	frame 2	.LLGAIIGLMVGGVVIATVIVITLV..
	frame 3	LLLGAIIGLMVGGVVIATVIVITL...
**Notch 1 (NOTC1_human)**	NMR[1]	...PAQ**LHFMYVAAAAFVLLFFVGCGVLLS**RKRR...[1]
	frame 0	...FMYVAAAAFVLLFFVGCGVLL
	frame 1	..LFMYVAAAAFVLLFFVGCGVL.
	frame 2	.LLFMYVAAAAFVLLFFVGCGV..
	frame 3	LLLFMYVAAAAFVLLFFVGCG...
**ErbB4 (ERBB4_human)**	NMR[1]	...HART**PLIAAGVIGGLFILVIVGLTFAVYVRR**KSI...[1]
	frame 0	...LIAAGVIGGLFILVIVGLTFAVYV
	frame 1	..LLIAAGVIGGLFILVIVGLTFAVY.
	frame 2	.LLLIAAGVIGGLFILVIVGLTFAV..
	frame 3	LLLLIAAGVIGGLFILVIVGLTFA...
**N-Cadherin (CADH2_human)**	predicted[3]	...LGTGA**IIAILLCIIILLILVLMFVVW**MKRRD... [3]
	frame 0	...GAIIAILLCIIILLILVLMFVVW
	frame 1	..LGAIIAILLCIIILLILVLMFVV.
	frame 2	.LLGAIIAILLCIIILLILVLMFV..
	frame 3	LLLGAIIAILLCIIILLILVLMF...
**Integrin β1 (ITB1_human)**	predicted[3]	...PTGPD**IIPIVAGVVAGIVLIGLALLLIW**KLLMI... [3]
	frame 0	...IIPIVAGVVAGIVLIGLALLLIW
	frame 1	..LIIPIVAGVVAGIVLIGLALLLI.
	frame 2	.LLIIPIVAGVVAGIVLIGLALLL..
	frame 3	LLLIIPIVAGVVAGIVLIGLALL...
**Nicastrin** [4]	frame 0	...LITLTVGFGILIFSLIVTY
	frame 1	..LLITLTVGFGILIFSLIVT.
	frame 2	.LLLITLTVGFGILIFSLIV..
	frame 3	LLLLITLTVGFGILIFSLI...
**PEN-2** [4]	frame 0	...AVGFLFWVIVLTSWITIFQIY
	frame 1	..LAVGFLFWVIVLTSWITIFQI.
	frame 2	.LLAVGFLFWVIVLTSWITIFQ..
	frame 3	LLLAVGFLFWVIVLTSWITIF...
**PS1 TMD1** [4]	frame 0	HVIMLFVPVTLCMVVVVATI...
	frame 1	.VIMLFVPVTLCMVVVVATIL..
	frame 2	..IMLFVPVTLCMVVVVATILL.
	frame 3	...MLFVPVTLCMVVVVATILLL
**PS1 TMD2** [4]	frame 0	...ILNAAIMISVIVVMTILLVVLY
	frame 1	..LILNAAIMISVIVVMTILLVVL.
	frame 2	.LLILNAAIMISVIVVMTILLVV..

	frame 3	LLLILNAAIMISVIVVMTILLV...
**PS1 TMD3**[4 5]	frame 0	VIHAWLIISSLLLLFFFSFIYLG…
	frame 1	.IHAWLIISSLLLLFFFSFIYLGL..
	frame 2	..HAWLIISSLLLLFFFSFIYLGLL.
	frame 3	...AWLIISSLLLLFFFSFIYLGLLL
**PS1 TMD4** [4]	frame 0	...YITVALLIWNFGVVGMISIHW
	frame 1	..LYITVALLIWNFGVVGMISIH.
	frame 2	.LLYITVALLIWNFGVVGMISI..
	frame 3	LLLYITVALLIWNFGVVGMIS...
**PS1 TMD5** [4]	frame 0	LRLQQAYLIMISALMALVFI...
	frame 1	.RLQQAYLIMISALMALVFIL..
	frame 2	..LQQAYLIMISALMALVFILL.
	frame 3	...QQAYLIMISALMALVFILLL
**PS1 TMD6** [4]	frame 0	...WTAWLILAVISVYDLVAVLCP
	frame 1	..LWTAWLILAVISVYDLVAVLC.
	frame 2	.LLWTAWLILAVISVYDLVAVL..
	frame 3	LLLWTAWLILAVISVYDLVAV...
**PS1 TMD7 D385L/K395L** [4,6]	frame 0	LGLGLFIFYSVLVGLASA...
	frame 1	.GLGLFIFYSVLVGLASAL..
	frame 2	..LGLFIFYSVLVGLASALL.
	frame 3	...GLFIFYSVLVGLASALLL
**PS1 TMD8** [4]	frame 0	...NTTIACFVAILIGLCLTLLLLA
	frame 1	..LNTTIACFVAILIGLCLTLLLL.
	frame 2	.LLNTTIACFVAILIGLCLTLLL..
	frame 3	LLLNTTIACFVAILIGLCLTLL...
**PS1 TMD9 D450L** [4, 5,6]	frame 0	PALPISITFGLVFYFATLYLV...
	frame 1	.ALPISITFGLVFYFATLYLVL..
	frame 2	..LPISITFGLVFYFATLYLVLL.
	frame 3	...PISITFGLVFYFATLYLVLLL
**GpA**		LIIFGVMAGVIGTILLISY
**GpA G83I** [8]		LIIFGVMAIVIGTILLISY
**EmrE TMD4**		LPAIIGMMLICAGVLIINL
**EmrE TMD4 G90V/G97V** [8]		LPAIIVMMLICAVVLIINL
**L20**		LLLLLLLLLLLLLLLLLLLL

[1] TMDs where NMR spectroscopy had previously identified the helical parts, as given in bold face (APP, pdb 6HYF; Notch1, pdb 8OR5; ErbB4, pdb 2LCX). In general, juxtamembrane domains were omitted to prevent the potential interference of their polar residues with membrane insertion and/or transmembrane topology. An invariant cytoplasmically located Lys or Arg residue acts as stop-transfer signal in N_{out} and N_{in} constructs.
[2] Frames 0 – 3 were tested for heteromerization. The stepwise insertion of up to three additional residues at a given N-terminus concurrent with the stepwise deletion of three residues at the C-terminus rotates potential TMD-TMD interfaces by up to 3 × 100°, i.e., almost a full helix turn, relative to the soluble domains in the hybrid BLa proteins.
[3] TMDs where the helical parts had been predicted from the sequences, as annotated in UniProtKB.

[4] TMDs from γ–secretase subunits, as identified in the cryo-EM structure (pdb 5fn2 {Bai, 2015 #7886}) except for the exceptionally long PS1 TMD2 where the sequence shown here corresponds to the length yielding the maximal LD_{50} (see: Fig. 2).
[5] PS1 TMD3 is truncated right before E184 as not to interfere with membrane integration.
[6] Residues marked in red indicate point mutations of ionizable residues that were necessary to integrate the TMD into the membrane.
[7] TMD9 is truncated after V453 where a helix kink occurs in the structure.
[8] Residues marked in red indicate point mutations at the respective helix-helix interfaces reducing the affinities of self-interaction.

Table S2. Sequences and frames of all TMDs used in the library screening approach (approach 2).

**A4_HUMAN**	frame 0	...GAIIGLMVGGVVIATVIVITLVML
	frame 1	..LGAIIGLMVGGVVIATVIVITLVM.
	frame 2	.LLGAIIGLMVGGVVIATVIVITLV..
	frame 3	LLLGAIIGLMVGGVVIATVIVITL...
**ANPRC_HUMAN**	frame 0	...SAVTGIVVGALLGAGLLMAFYFF
	frame 1	..LSAVTGIVVGALLGAGLLMAFYF.
	frame 2	.LLSAVTGIVVGALLGAGLLMAFY..
	frame 3	LLLSAVTGIVVGALLGAGLLMAF...
**APLP1_HUMAN**	frame 0	...AVSGLLIMGAGGGSLIVLSMLLL
	frame 1	..LAVSGLLIMGAGGGSLIVLSMLL.
	frame 2	.LLAVSGLLIMGAGGGSLIVLSML..
	frame 3	LLLAVSGLLIMGAGGGSLIVLSM...
**APLP2_HUMAN**	frame 0	...ALIGLLVIAVAIATVIVISLVML
	frame 1	..LALIGLLVIAVAIATVIVISLVM.
	frame 2	.LLALIGLLVIAVAIATVIVISLV..
	frame 3	LLLALIGLLVIAVAIATVIVISL...
**CADH2_HUMAN**	frame 0	...GAIIAILLCIIILLILVLMFVVW
	frame 1	..LGAIIAILLCIIILLILVLMFVV.
	frame 2	.LLGAIIAILLCIIILLILVLMFV..
	frame 3	LLLGAIIAILLCIIILLILVLMF...
**CD44_HUMAN**	frame 0	...WLIIILASLLALALILAVCIAV
	frame 1	..LWLIIILASLLALALILAVCIA.
	frame 2	.LLWLIIILASLLALALILAVCI..
	frame 3	LLLWLIIILASLLALALILAVC...
**CSF1R_HUMAN**	frame 0	...VVVACMSIMALLLLLLLLLLY
	frame 1	..LVVVACMSIMALLLLLLLLLL.
	frame 2	.LLVVVACMSIMALLLLLLLLL..
	frame 3	LLLVVVACMSIMALLLLLLLL...
**CSTN1_HUMAN**	frame 0	...ATVVIVVCVSFLVFMIILGVF
	frame 1	..LATVVIVVCVSFLVFMIILGV.
	frame 2	.LLATVVIVVCVSFLVFMIILG..
	frame 3	LLLATVVIVVCVSFLVFMIIL...
**CSTN2_HUMAN**	frame 0	...IATVVIIISVCMLVFVVAMGV
	frame 1	..LIATVVIIISVCMLVFVVAMG.
	frame 2	.LLIATVVIIISVCMLVFVVAM..
	frame 3	LLLIATVVIIISVCMLVFVVA...
**DCC_HUMAN**	frame 0	...LLVIIVVTVGVITVLVVVIVAVI
	frame 1	..LLLVIIVVTVGVITVLVVVIVAV.
	frame 2	.LLLLVIIVVTVGVITVLVVVIVA..
	frame 3	LLLLLVIIVVTVGVITVLVVVIV...

**DNER_HUMAN**	frame 0	...IIIGALCVAFILMLIILIVGI
	frame 1	..LIIIGALCVAFILMLIILIVG.
	frame 2	.LLIIIGALCVAFILMLIILIV..
	frame 3	LLLIIIGALCVAFILMLIILI...
**DSG2_HUMAN**	frame 0	...PAAIALMILAFLLLLLVPLLLLM
	frame 1	..LPAAIALMILAFLLLLLVPLLLL.
	frame 2	.LLPAAIALMILAFLLLLLVPLLL..
	frame 3	LLLPAAIALMILAFLLLLLVPLL...
**ERBB4_HUMAN**	frame 0	...LIAAGVIGGLFILVIVGLTFAVYV
	frame 1	..LLIAAGVIGGLFILVIVGLTFAVY.
	frame 2	.LLLIAAGVIGGLFILVIVGLTFAV..
	frame 3	LLLLIAAGVIGGLFILVIVGLTFA...
**GLPA_HUMAN**	frame 0	...ITLIIFGVMAGVIGTILLISYGI
	frame 1	..LITLIIFGVMAGVIGTILLISYG.
	frame 2	.LLITLIIFGVMAGVIGTILLISY..
	frame 3	LLLITLIIFGVMAGVIGTILLIS...
**HLAA_HUMAN**	frame 0	...VGIIAGLVLLGAVITGAVVAAVMW
	frame 1	..LVGIIAGLVLLGAVITGAVVAAVM.
	frame 2	.LLVGIIAGLVLLGAVITGAVVAAV..
	frame 3	LLLVGIIAGLVLLGAVITGAVVAA...
**ITB1_HUMAN**	frame 0	...IIPIVAGVVAGIVLIGLALLLIW
	frame 1	..LIIPIVAGVVAGIVLIGLALLLI.
	frame 2	.LLIIPIVAGVVAGIVLIGLALLL..
	frame 3	LLLIIPIVAGVVAGIVLIGLALL...
**JAG2_HUMAN**	frame 0	...GLLVPVLCGAFSVLWLACVVL
	frame 1	..LGLLVPVLCGAFSVLWLACVV.
	frame 2	.LLGLLVPVLCGAFSVLWLACV..
	frame 3	LLLGLLVPVLCGAFSVLWLAC...
**LDLR_HUMAN**	frame 0	...ALSIVLPIVLLVFLCLGVFLLW
	frame 1	..LALSIVLPIVLLVFLCLGVFLL.
	frame 2	.LLALSIVLPIVLLVFLCLGVFL..
	frame 3	LLLALSIVLPIVLLVFLCLGVF...
**NOTC1_HUMAN**	frame 0	...FMYVAAAAFVLLFFVGCGVLL
	frame 1	..LFMYVAAAAFVLLFFVGCGVL.
	frame 2	.LLFMYVAAAAFVLLFFVGCGV..
	frame 3	LLLFMYVAAAAFVLLFFVGCG...
**NOTC2_HUMAN**	frame 0	...LLYLLAVAVVIILFIILLGVI
	frame 1	..LLLYLLAVAVVIILFIILLGV.
	frame 2	.LLLLYLLAVAVVIILFIILLG..
	frame 3	LLLLLYLLAVAVVIILFIILL...
**NOTC3_HUMAN**	frame 0	...LLPLLVAGAVLLLVILVLGVMVA
	frame 1	..LLLPLLVAGAVLLLVILVLGVMV.

	frame 2	.LLLLPLLVAGAVLLLVILVLGVM..
	frame 3	LLLLLPLLVAGAVLLLVILVLGV...
**NOTC4_HUMAN**	frame 0	...VLCSPVAGVILLALGALLVLQLI
	frame 1	..LVLCSPVAGVILLALGALLVLQL.
	frame 2	.LLVLCSPVAGVILLALGALLVLQ..
	frame 3	LLLVLCSPVAGVILLALGALLVL...
**PTPRT_HUMAN**	frame 0	...MAGVIAGLLMFIIILLGVMLTI
	frame 1	..LMAGVIAGLLMFIIILLGVMLT.
	frame 2	.LLMAGVIAGLLMFIIILLGVML..
	frame 3	LLLMAGVIAGLLMFIIILLGVM...
**PXDC2_HUMAN**	frame 0	...GLIIGILILVLIVATAILVTV
	frame 1	..LGLIIGILILVLIVATAILVT.
	frame 2	.LLGLIIGILILVLIVATAILV..
	frame 3	LLLGLIIGILILVLIVATAIL...
**SDC1_HUMAN**	frame 0	...GVIAGGLVGLIFAVCLVGFML
	frame 1	..LGVIAGGLVGLIFAVCLVGFM.
	frame 2	.LLGVIAGGLVGLIFAVCLVGF..
	frame 3	LLLGVIAGGLVGLIFAVCLVG...
**SDC2_HUMAN**	frame 0	...VLAAVIAGGVIGFLFAIFLILLLV
	frame 1	..LVLAAVIAGGVIGFLFAIFLILLL.
	frame 2	.LLVLAAVIAGGVIGFLFAIFLILL..
	frame 3	LLLVLAAVIAGGVIGFLFAIFLIL...
**TNR17_HUMAN**	frame 0	...ILWTCLGLSLIISLAVFVLMFLL
	frame 1	..LILWTCLGLSLIISLAVFVLMFL.
	frame 2	.LLILWTCLGLSLIISLAVFVLMF..
	frame 3	LLLILWTCLGLSLIISLAVFVLM...
**TYOBP_HUMAN**	frame 0	...GVLAGIVMGDLVLTVLIALAV
	frame 1	..LGVLAGIVMGDLVLTVLIALA.
	frame 2	.LLGVLAGIVMGDLVLTVLIAL..
	frame 3	LLLGVLAGIVMGDLVLTVLIA...
**PS1 TMD2**	cryo-EM[1]	...VGQRALHSILNAAIMISVIVVMTILLVVLY
	frame 3	LLLVGQRALHSILNAAIMISVI...........
	frame 2	.LLVGQRALHSILNAAIMISVIV..........
	frame 1	..LVGQRALHSILNAAIMISVIVV.........
	frame 0	...VGQRALHSILNAAIMISVIVVM........
	frame -1	....GQRALHSILNAAIMISVIVVMT.......
	frame -2	.....QRALHSILNAAIMISVIVVMTI......
	frame -3	......RALHSILNAAIMISVIVVMTIL.....
	frame -4	.......ALHSILNAAIMISVIVVMTILL....
	frame -5	........LHSILNAAIMISVIVVMTILLV...
	frame -6	.........HSILNAAIMISVIVVMTILLVV..
	frame -7	..........SILNAAIMISVIVVMTILLVVL.

frame -8	...........ILNAAIMISVIVVMTILLVVLY
**GpA**	LIIFGVMAGVIGTILLISY
**GpA G83I 8**	LIIFGVMAIVIGTILLISY
**L20**	LLLLLLLLLLLLLLLLLLLL

[1] TMD2 from presenilin 1, as identified in the cryo-EM structure (pdb 5fn2 {Bai, 2015 #7886}).

Table S3. DNA oligonucleotide sequences used in qPCR and NGS.

**GpA wt control primer pair**	
**qPCR_NBLa_fwd[1]**	CAAACGACGAGCGTGACACC
**qPCR_GpA_wt_rev**	GAATGGTGCCAATCACGCC
**GpA G83I control primer pair**	
**qPCR_NBLa_fwd[1]**	CAAACGACGAGCGTGACACC
**qPCR_GpA_G83I_rev**	AGCAGAATGGTGCCAATCACAAT
**Full plasmid quantification control primer pair**	
**qPCR_plasmid_control_fwd**	CGAGAAGCAGGCCATTATCG
**qPCR_plasmid_control_fwd**	GATGGTCGTCATCTACCTGC
**Oligonucleotides used for NGS amplicon generation**	
**NBLa_NGS_fwd**	ACACTCTTTCCCTACACGACGCTCTTCCGATCT<u>GTAGCAATGGCAACAACG</u>[2]
**NBLa_NGS_rev**	GACTGGAGTTCAGACGTGTGCTCTTCCGATCTAATCCGAG<u>TAACCAGAATCGGAACAA</u>[2]
**Substrate library amplification**	
**[BIOTIN]BLa_Library_fwd**	[BIOTIN]GTTCTGCTAATCGAGCTAGC
**[BIOTIN]BLa_Library_rev**	[BIOTIN]TGCCAGTTTGTGGATCCC

[1] Shared oligonucleotide for both controls

[2] <u>underlined</u> sequences are priming regions within the NBLa-plasmid; 5’ parts are Illumina universal sequencing adapters

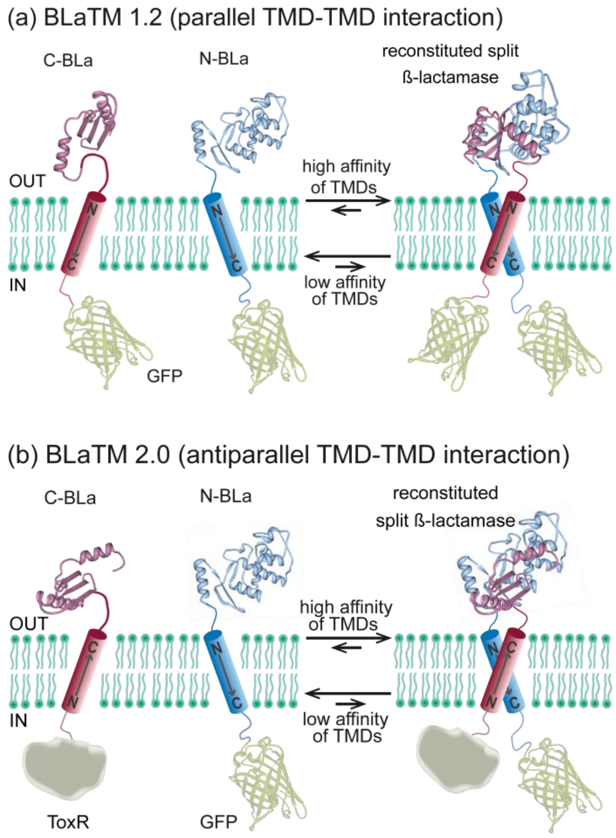

Figure S1. Design of the BLaTM system. **(a)** Scheme of BLaTM 1.2 used to study parallel TMD interactions (modified after Fig. 1 in 17). **(b)** Scheme of BLaTM 2.0 used to study antiparallel interactions. Reproduced, with permission, from ref. {Julius, 2017 #8260}.

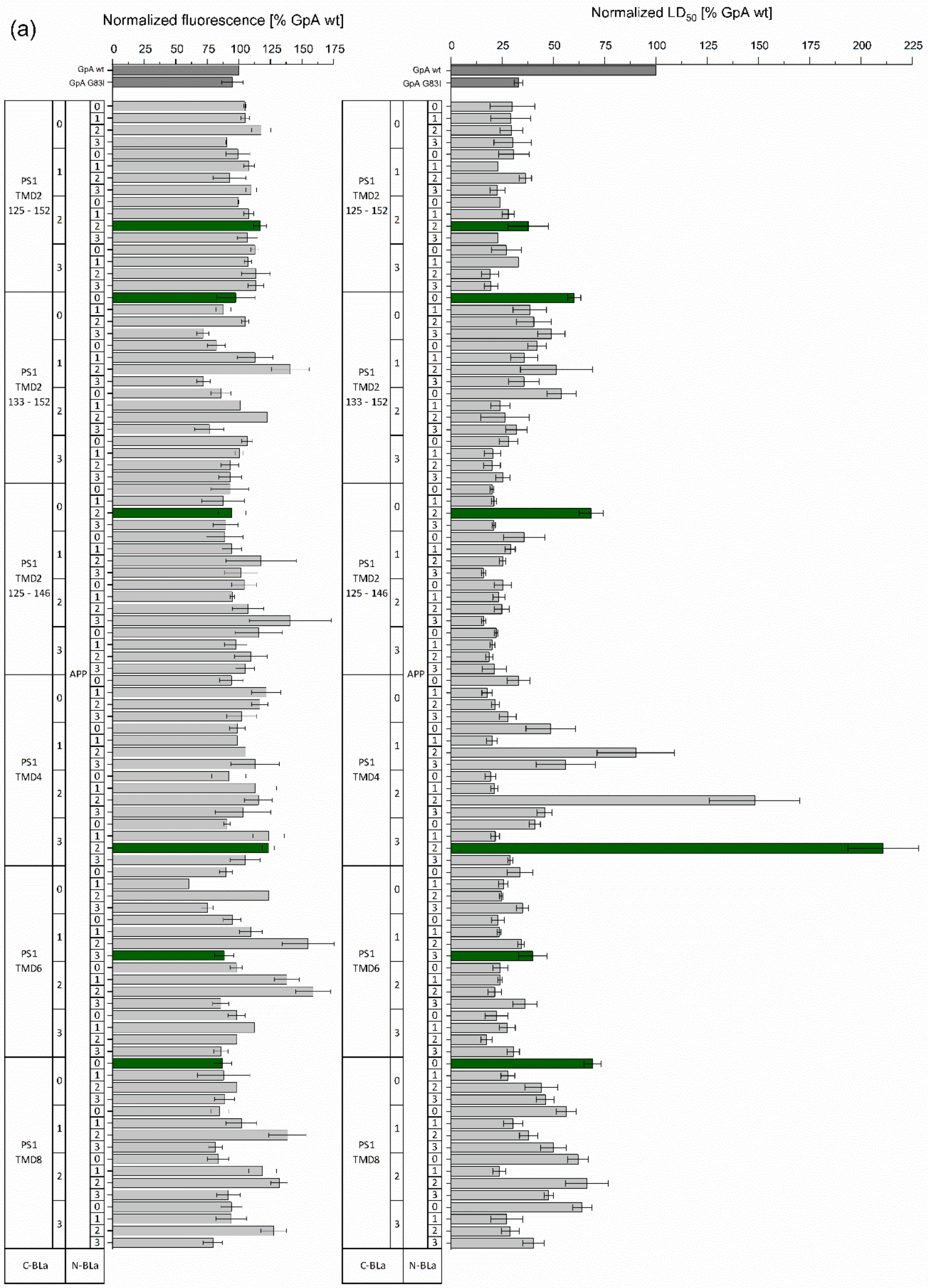
(a)
Normalized fluorescence [% GpA wt]
Normalized LD50 [% GpA wt]
GpA wt
GpA G83I
PS1 TMD2 125 - 152
PS1 TMD2 133 - 152
PS1 TMD2 125 - 146
PS1 TMD4
PS1 TMD6
PS1 TMD8
APP
C-BLa
N-BLa

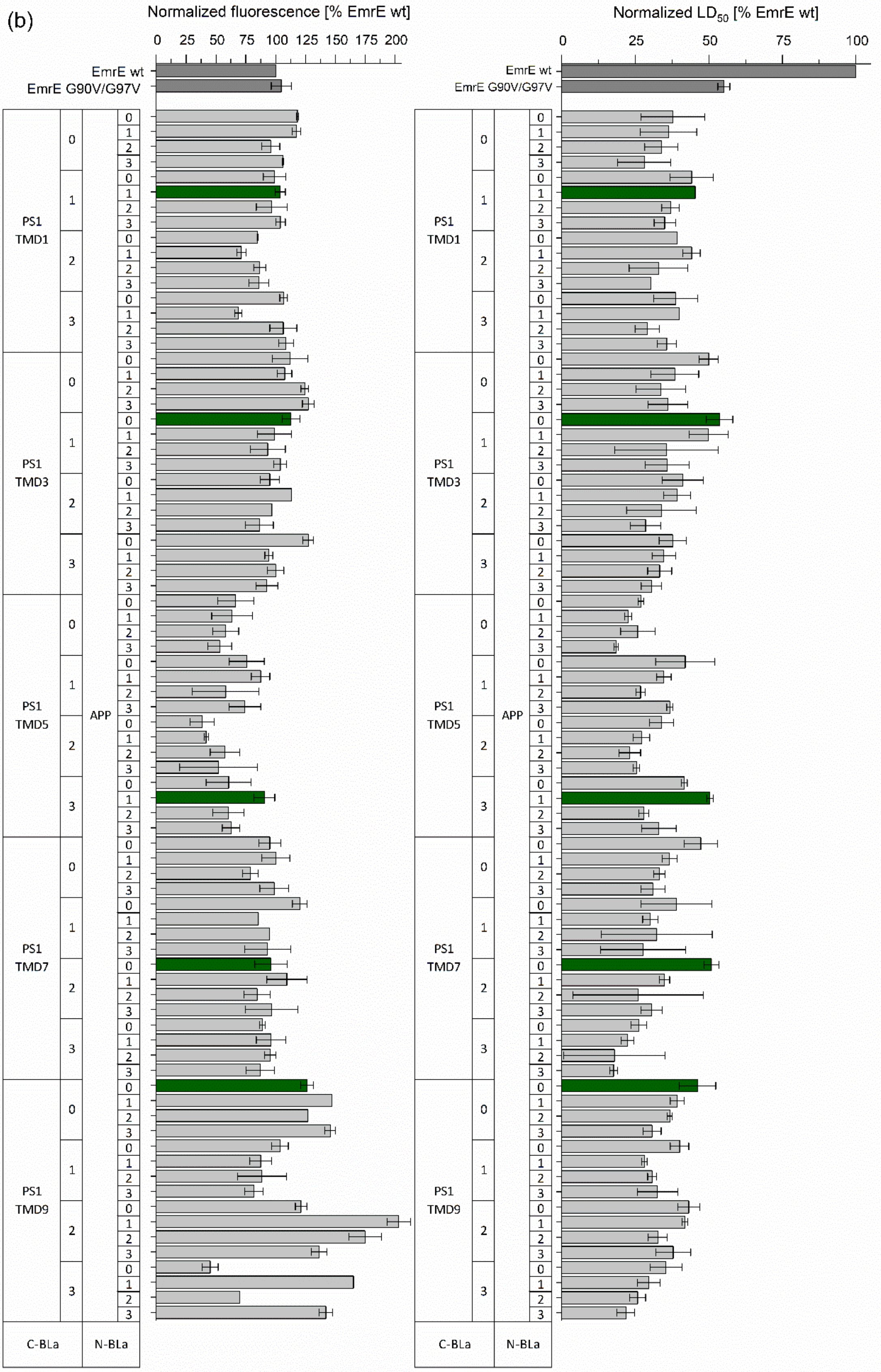

(b)
Normalized fluorescence [% EmrE wt]
0 25 50 75 100 125 150 175 200
Normalized LD$_{50}$ [% EmrE wt]
0 25 50 75 100
EmrE wt
EmrE G90V/G97V
PS1 TMD1
PS1 TMD3
PS1 TMD5
PS1 TMD7
PS1 TMD9
APP
C-BLa
N-BLa

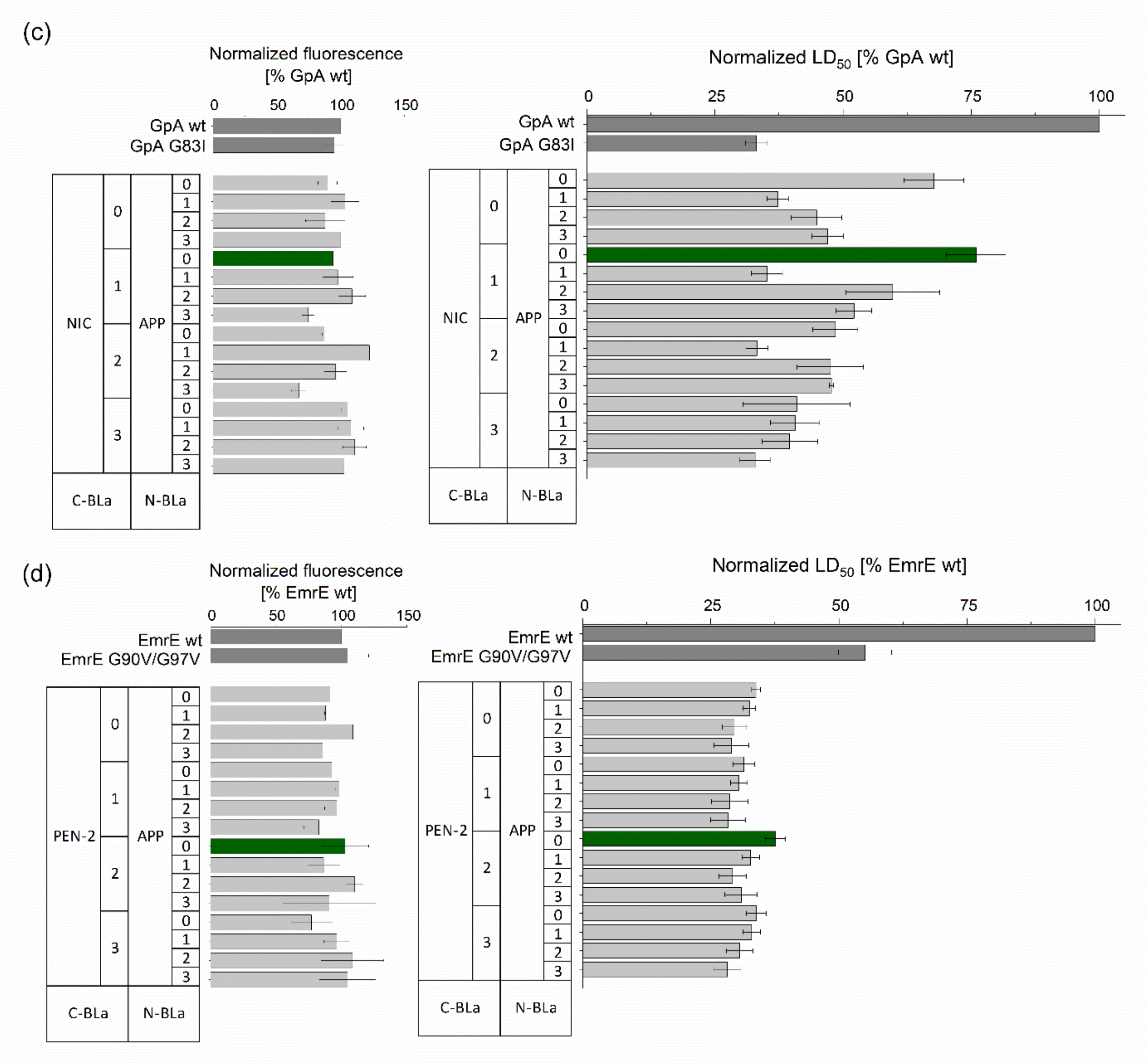
(c)
Normalized fluorescence [% GpA wt]
Normalized LD50 [% GpA wt]
GpA wt
GpA G83I
NIC
APP
C-BLa
N-BLa
(d)
Normalized fluorescence [% EmrE wt]
Normalized LD50 [% EmrE wt]
EmrE wt
EmrE G90V/G97V
PEN-2
APP
C-BLa
N-BLa

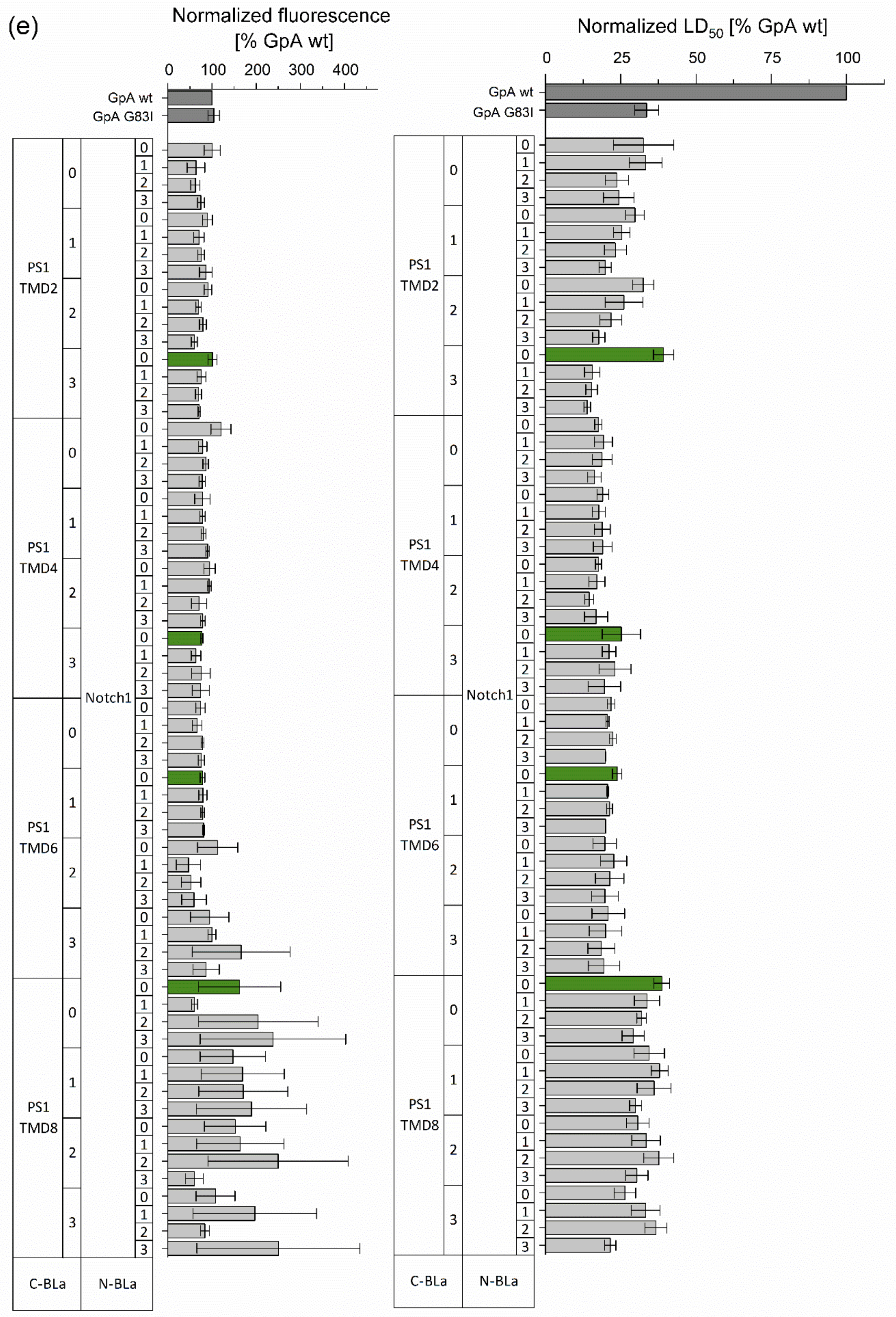

(e)
Normalized fluorescence [% GpA wt]
0
100
200
300
400
Normalized LD50 [% GpA wt]
0
25
50
75
100
GpA wt
GpA G83I
PS1 TMD2
PS1 TMD4
PS1 TMD6
PS1 TMD8
Notch1
C-BLa
N-BLa

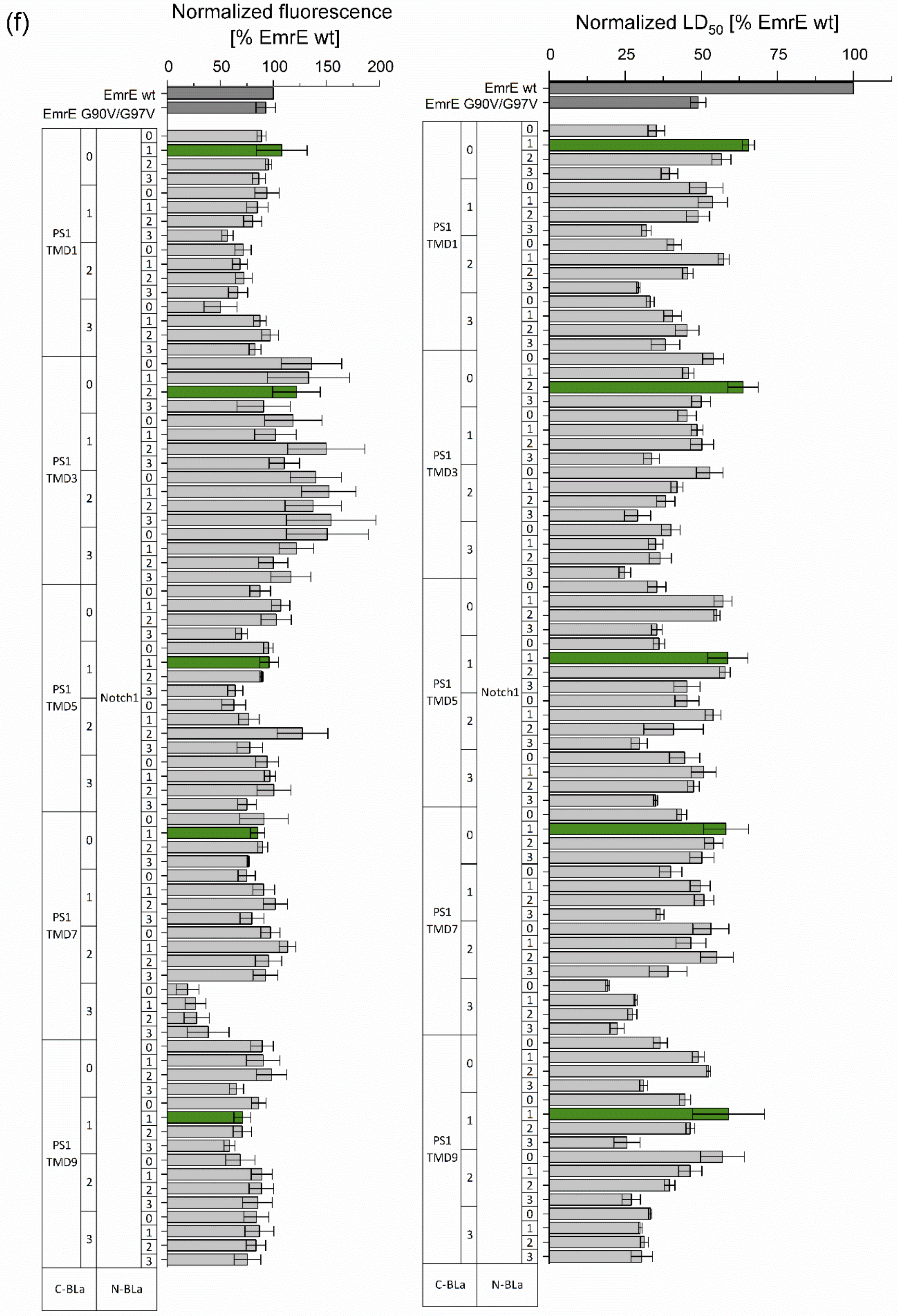
(f)
Normalized fluorescence [% EmrE wt]
0 50 100 150 200
EmrE wt
EmrE G90V/G97V
PS1 TMD1
PS1 TMD3
PS1 TMD5
PS1 TMD7
PS1 TMD9
Notch1
C-BLa
N-BLa
Normalized LD50 [% EmrE wt]
0 25 50 75 100

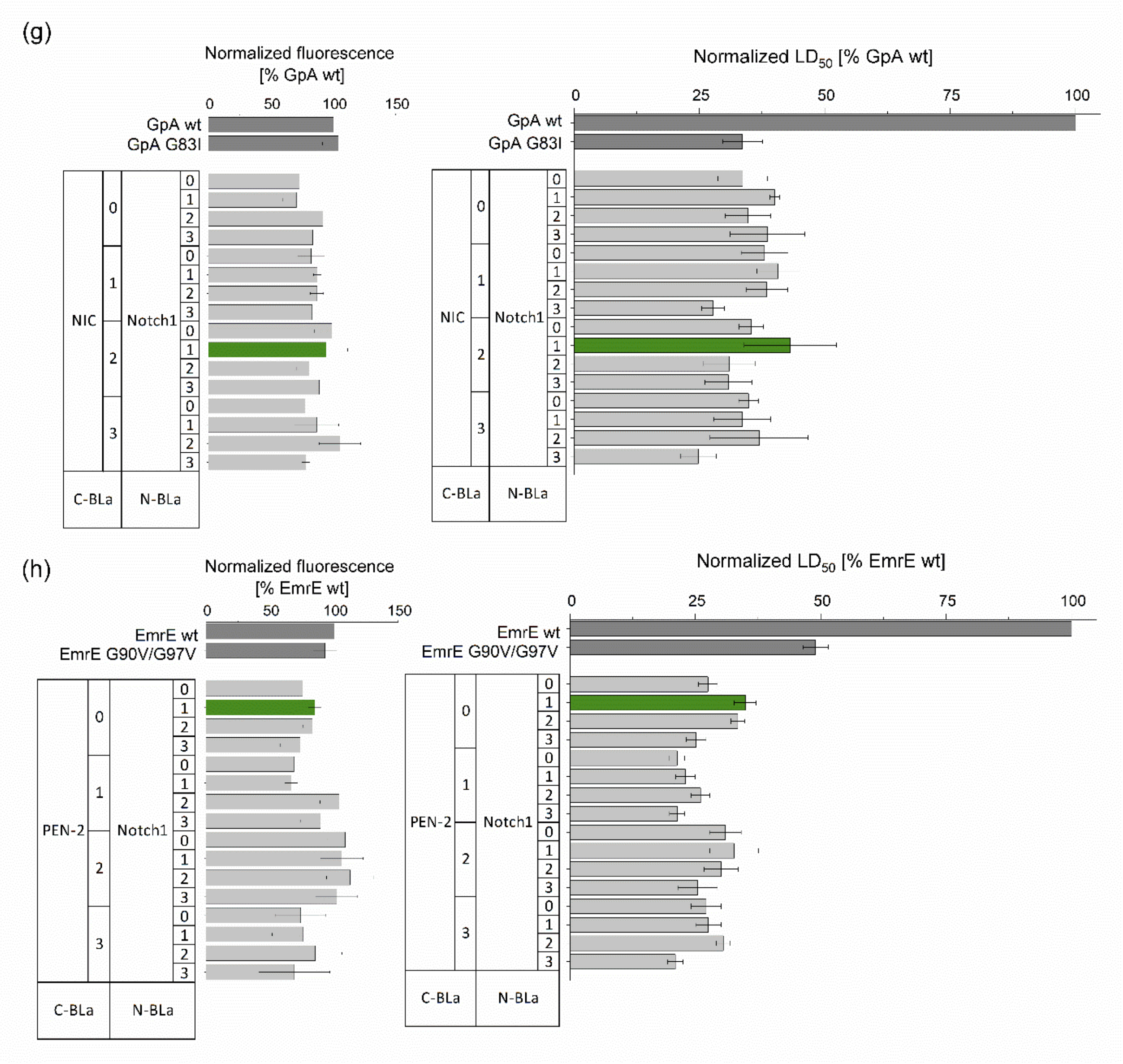
(g)
Normalized fluorescence [% GpA wt]
0 50 100 150
GpA wt
GpA G83I
NIC
Notch1
C-BLa
N-BLa
Normalized LD50 [% GpA wt]
0 25 50 75 100
(h)
Normalized fluorescence [% EmrE wt]
EmrE wt
EmrE G90V/G97V
PEN-2
Notch1
Normalized LD50 [% EmrE wt]

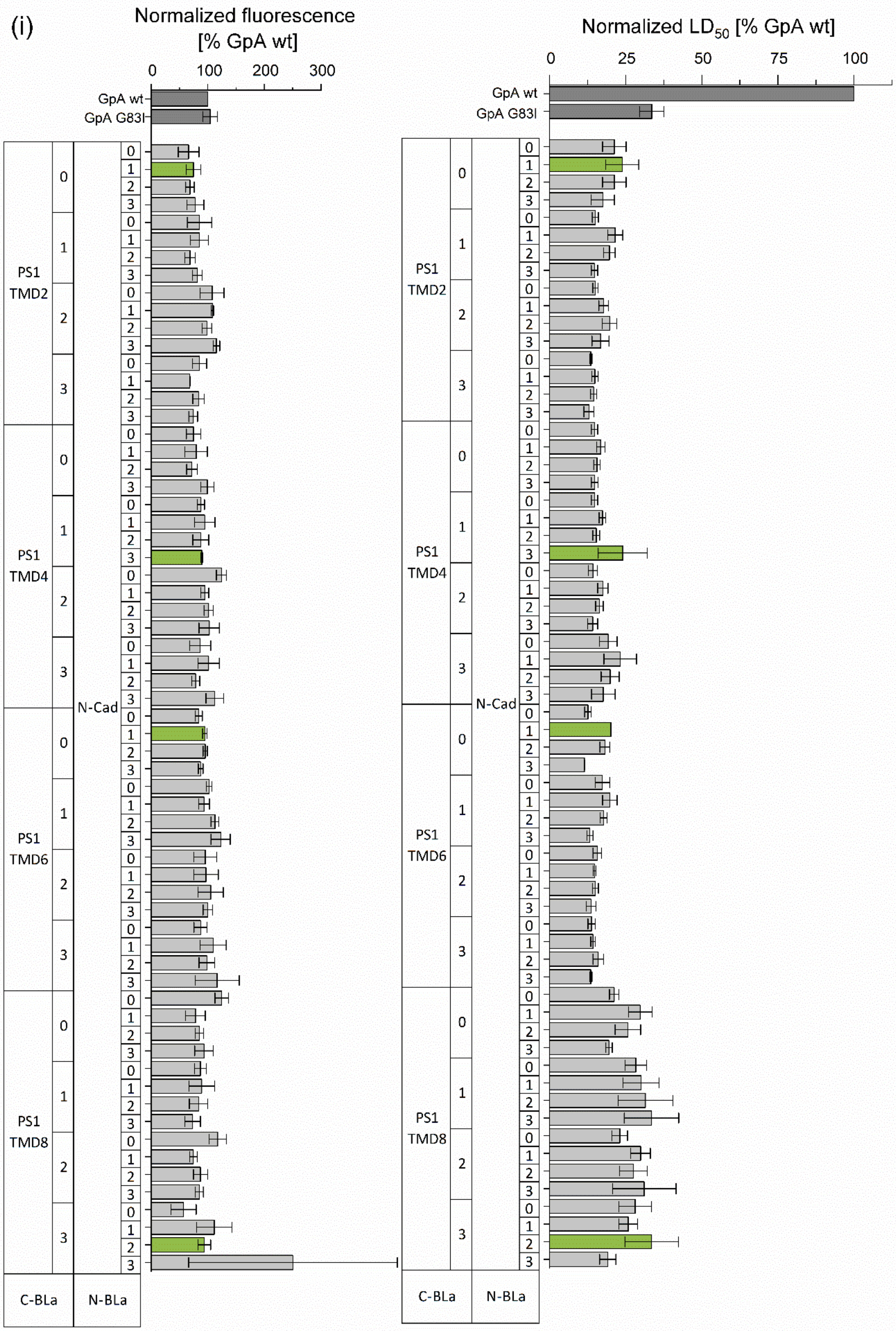
(i)
Normalized fluorescence [% GpA wt]
0
100
200
300
GpA wt
GpA G83I
PS1 TMD2
PS1 TMD4
PS1 TMD6
PS1 TMD8
N-Cad
C-BLa
N-BLa
Normalized LD50 [% GpA wt]
0
25
50
75
100

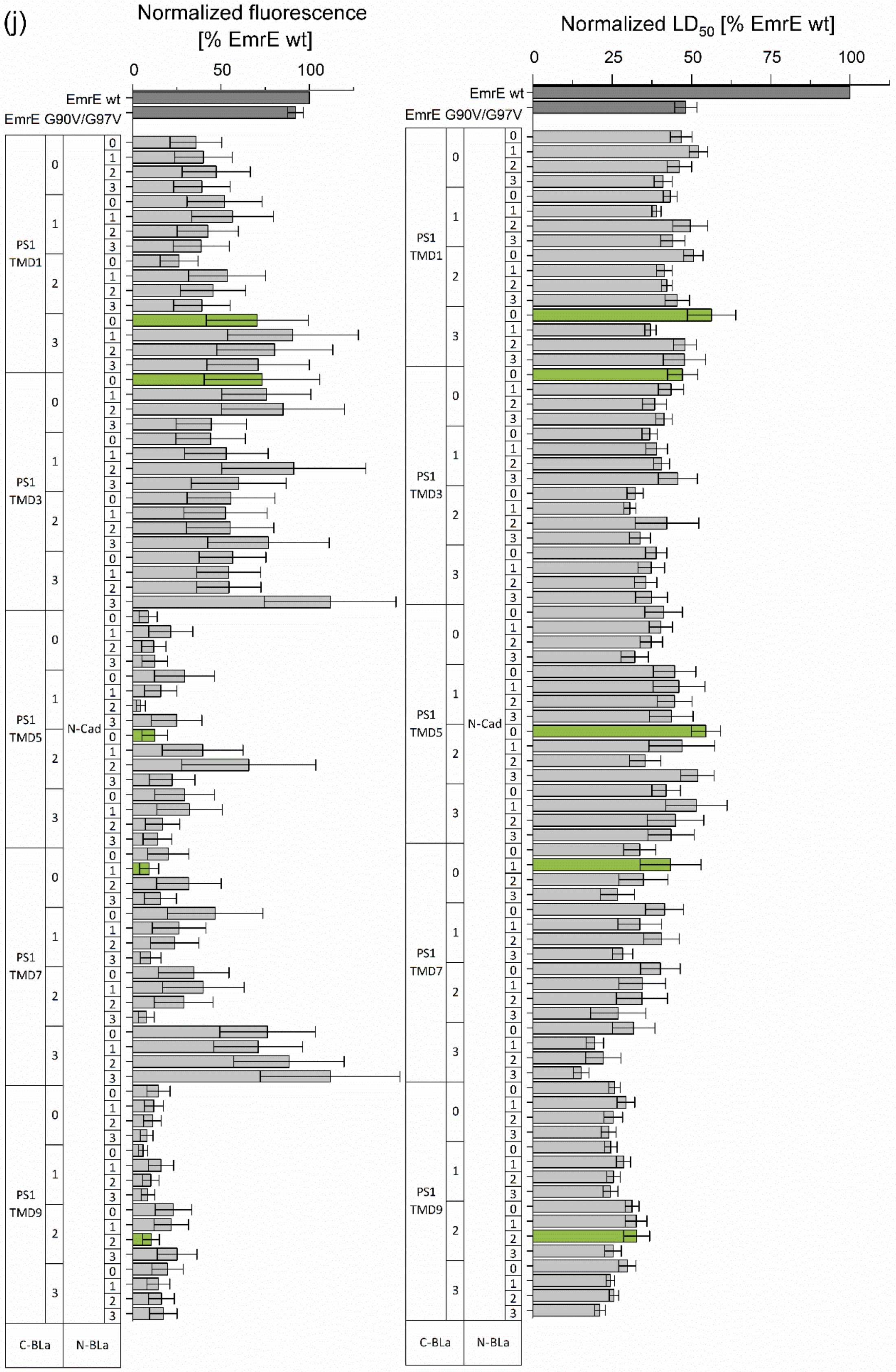

(j)
Normalized fluorescence [% EmrE wt]
0
50
100
EmrE wt
EmrE G90V/G97V
Normalized LD50 [% EmrE wt]
0
25
50
75
100
PS1 TMD1
PS1 TMD3
PS1 TMD5
PS1 TMD7
PS1 TMD9
N-Cad
C-BLa
N-BLa

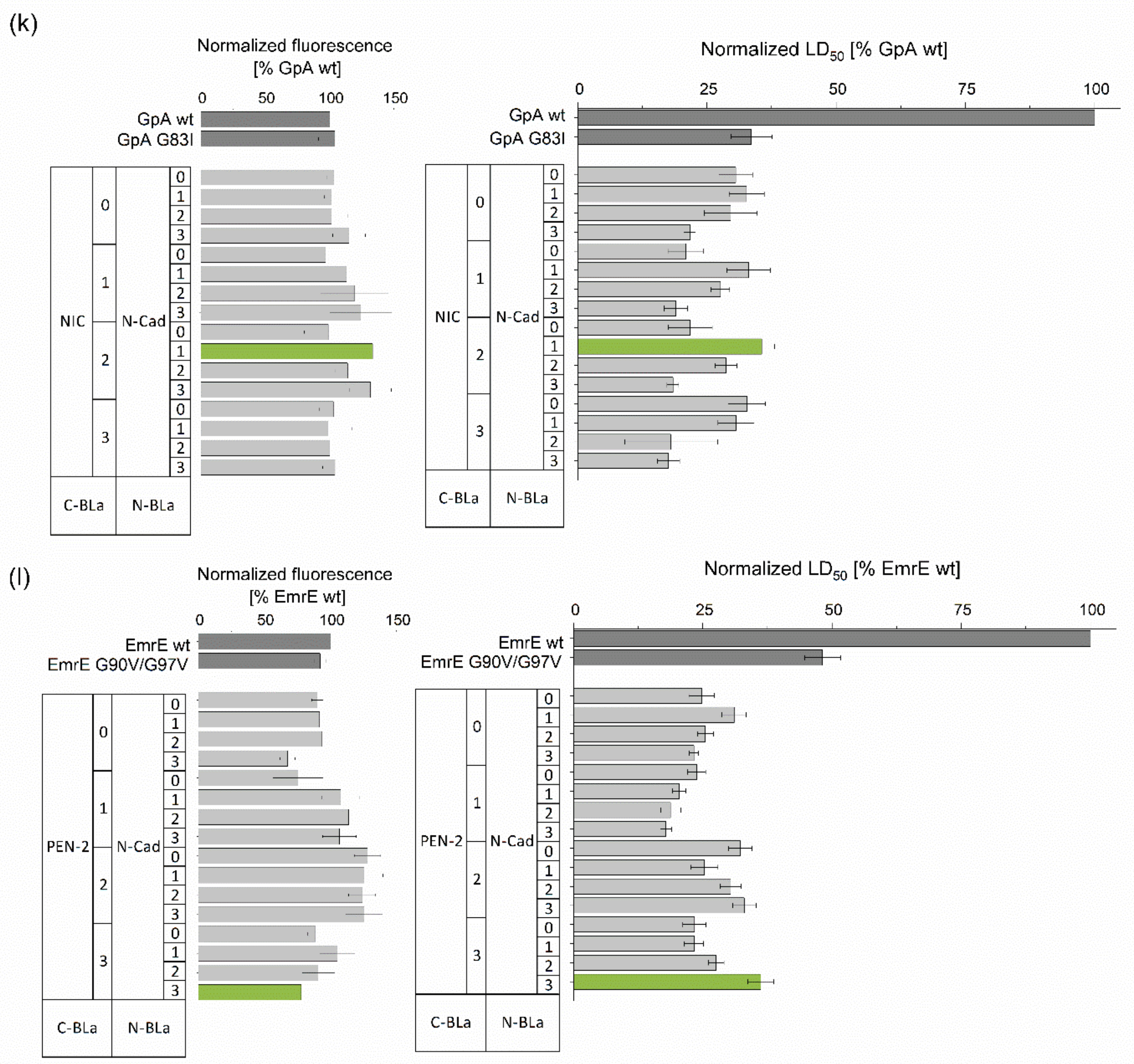
(k)
Normalized fluorescence
[% GpA wt]
0
50
100
150
GpA wt
GpA G83I
NIC
N-Cad
C-BLa
N-BLa
Normalized LD50 [% GpA wt]
0
25
50
75
100
(l)
Normalized fluorescence
[% EmrE wt]
EmrE wt
EmrE G90V/G97V
PEN-2
Normalized LD50 [% EmrE wt]

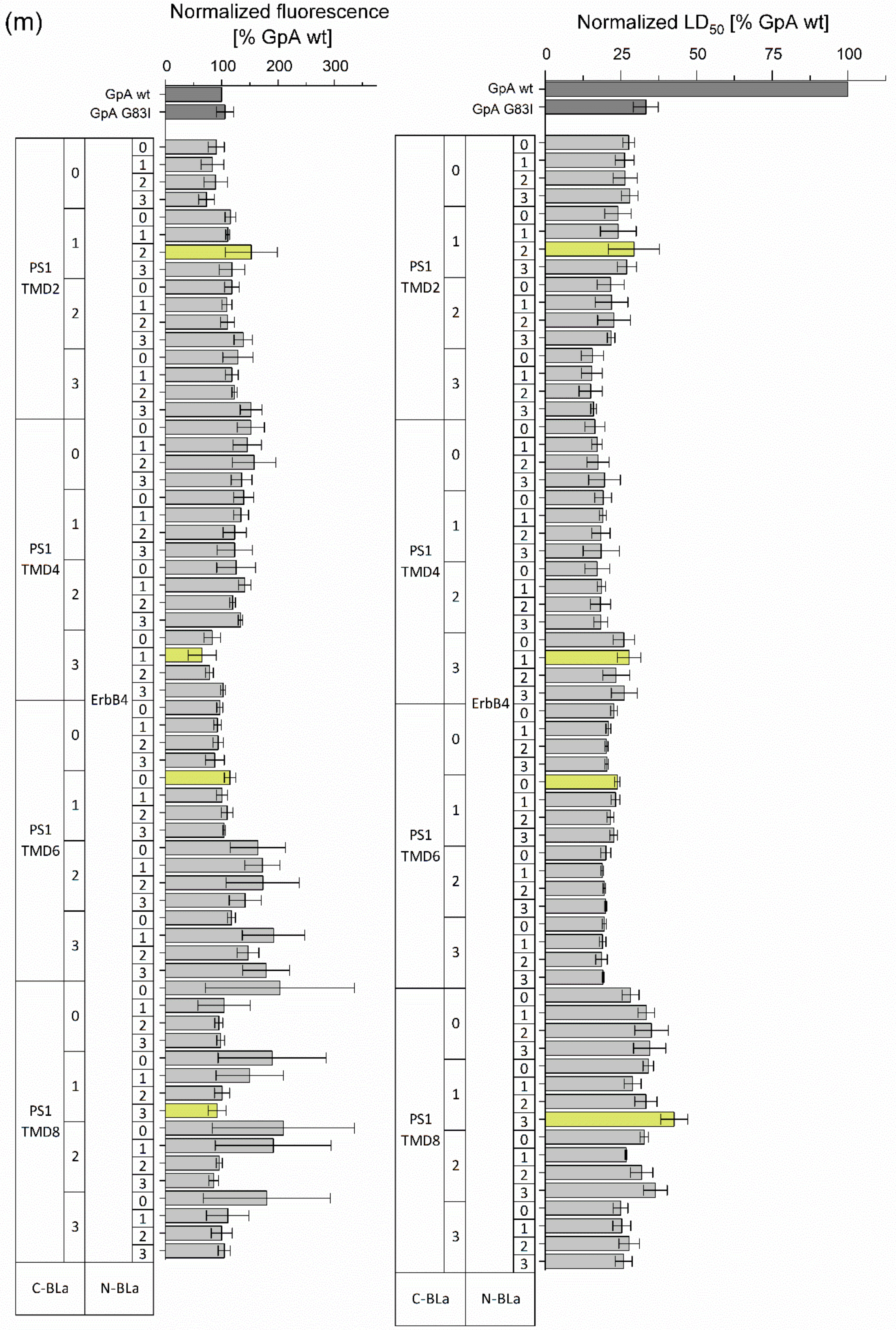

(m)
Normalized fluorescence [% GpA wt]
Normalized LD50 [% GpA wt]
GpA wt
GpA G83I
PS1 TMD2
PS1 TMD4
PS1 TMD6
PS1 TMD8
ErbB4
C-BLa
N-BLa

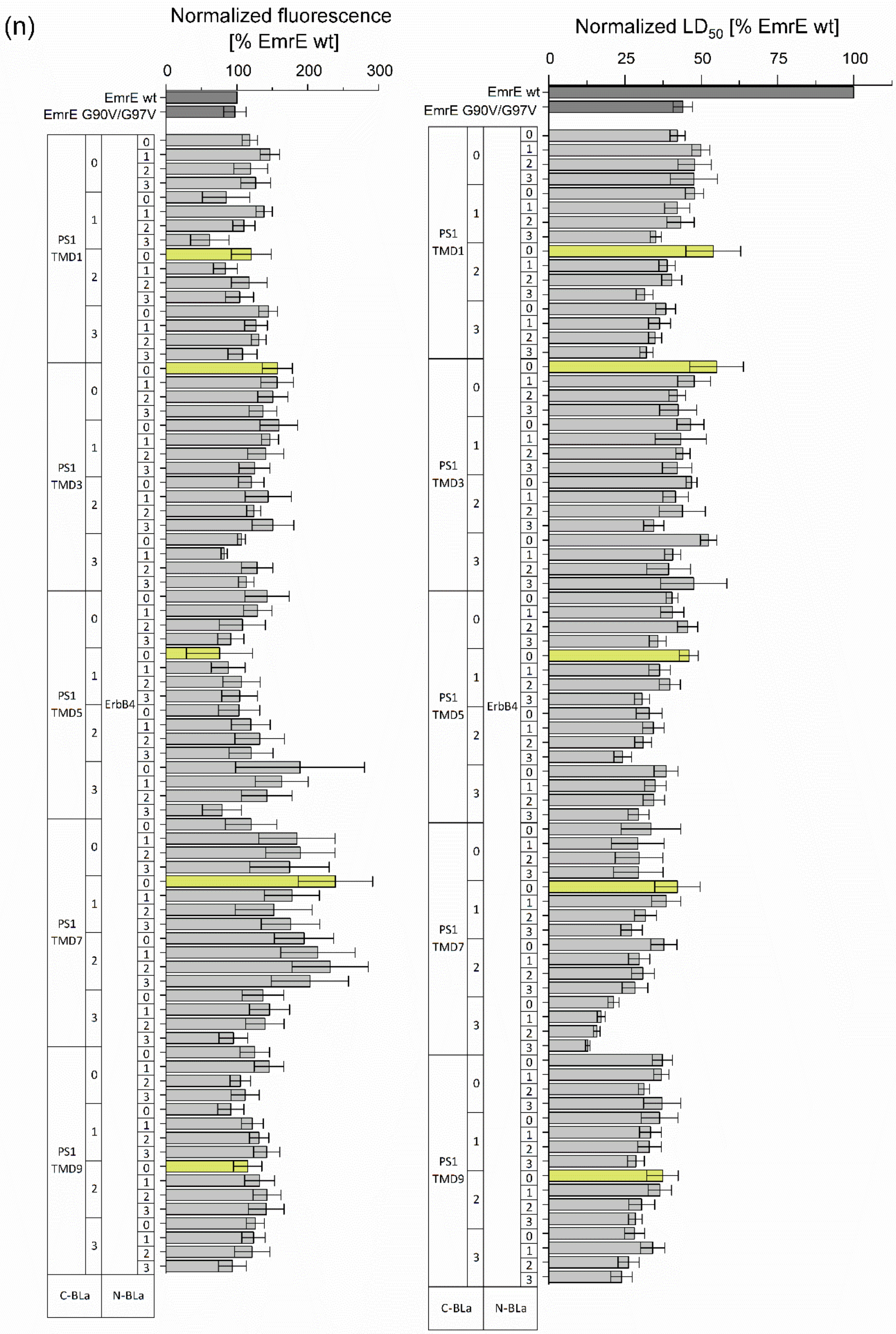
(n)
Normalized fluorescence [% EmrE wt]
0
100
200
300
EmrE wt
EmrE G90V/G97V
PS1 TMD1
PS1 TMD3
PS1 TMD5
PS1 TMD7
PS1 TMD9
ErbB4
C-BLa
N-BLa
Normalized LD50 [% EmrE wt]
0
25
50
75
100

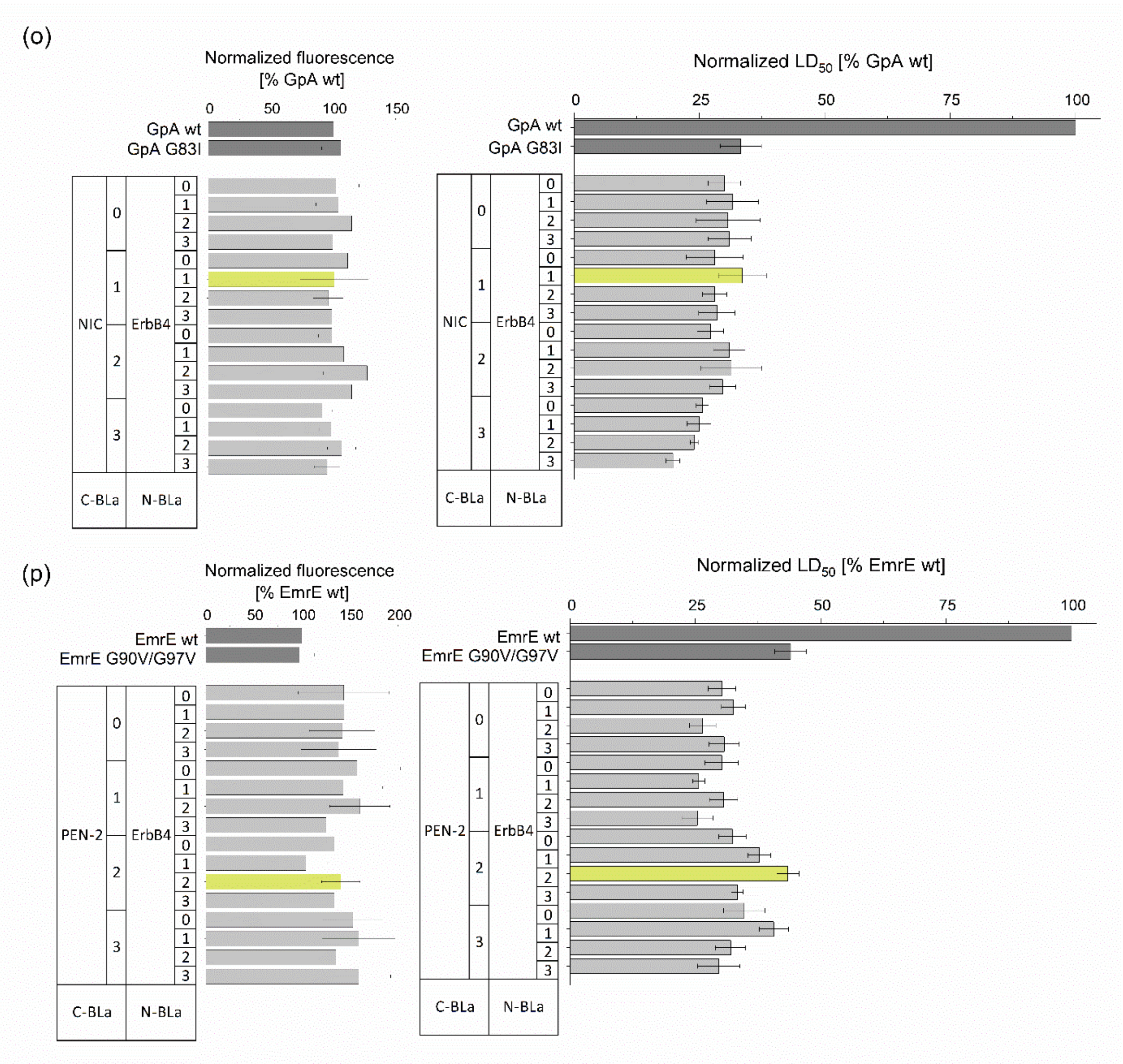

(o)
Normalized fluorescence [% GpA wt]
0 50 100 150
GpA wt
GpA G83I
NIC
ErbB4
C-BLa
N-BLa
Normalized LD50 [% GpA wt]
0 25 50 75 100
(p)
Normalized fluorescence [% EmrE wt]
0 50 100 150 200
EmrE wt
EmrE G90V/G97V
PEN-2
ErbB4
C-BLa
N-BLa
Normalized LD50 [% EmrE wt]
0 25 50 75 100

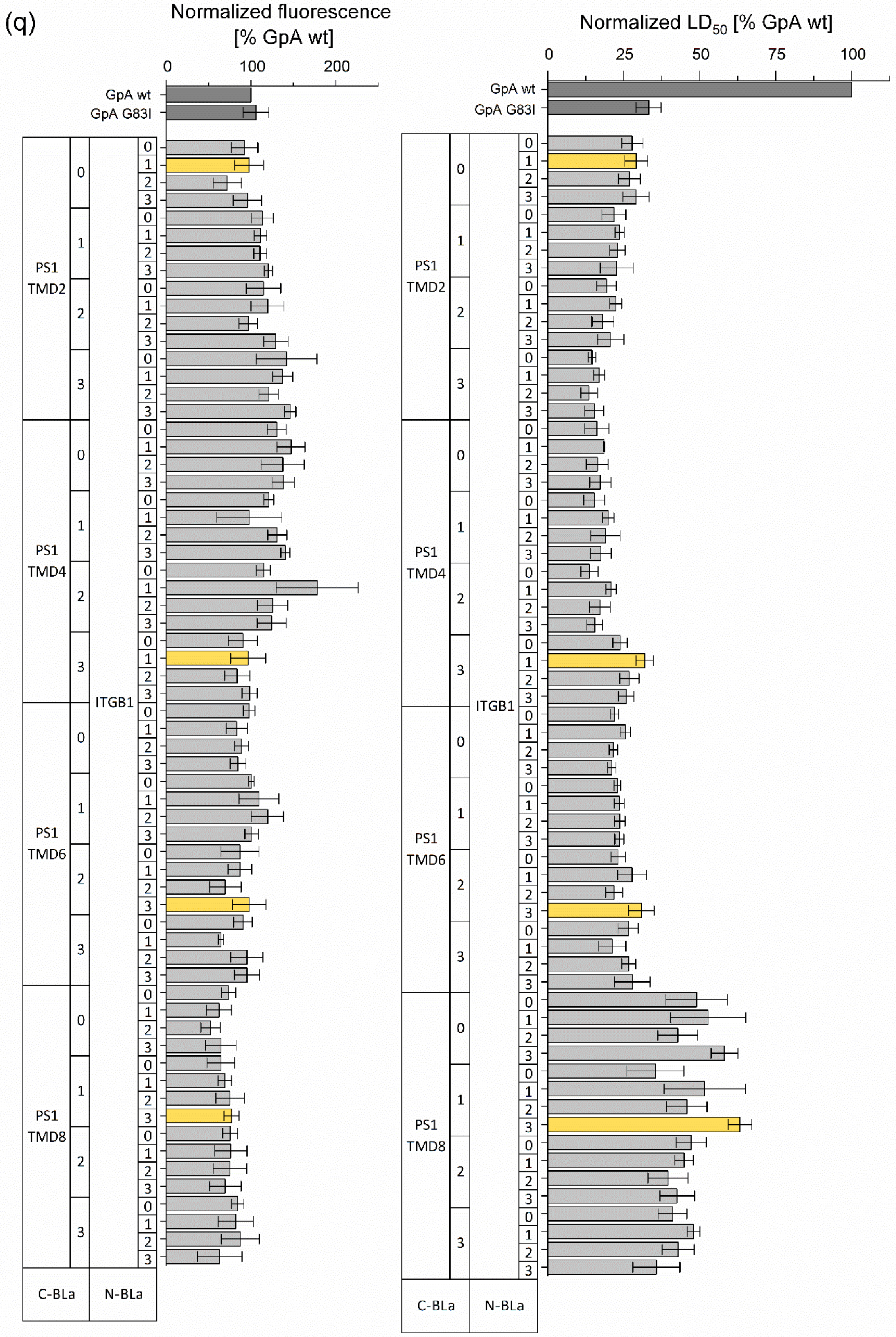
(q)
Normalized fluorescence [% GpA wt]
0
100
200
GpA wt
GpA G83I
PS1 TMD2
PS1 TMD4
PS1 TMD6
PS1 TMD8
ITGB1
C-BLa
N-BLa
Normalized LD50 [% GpA wt]
0
25
50
75
100

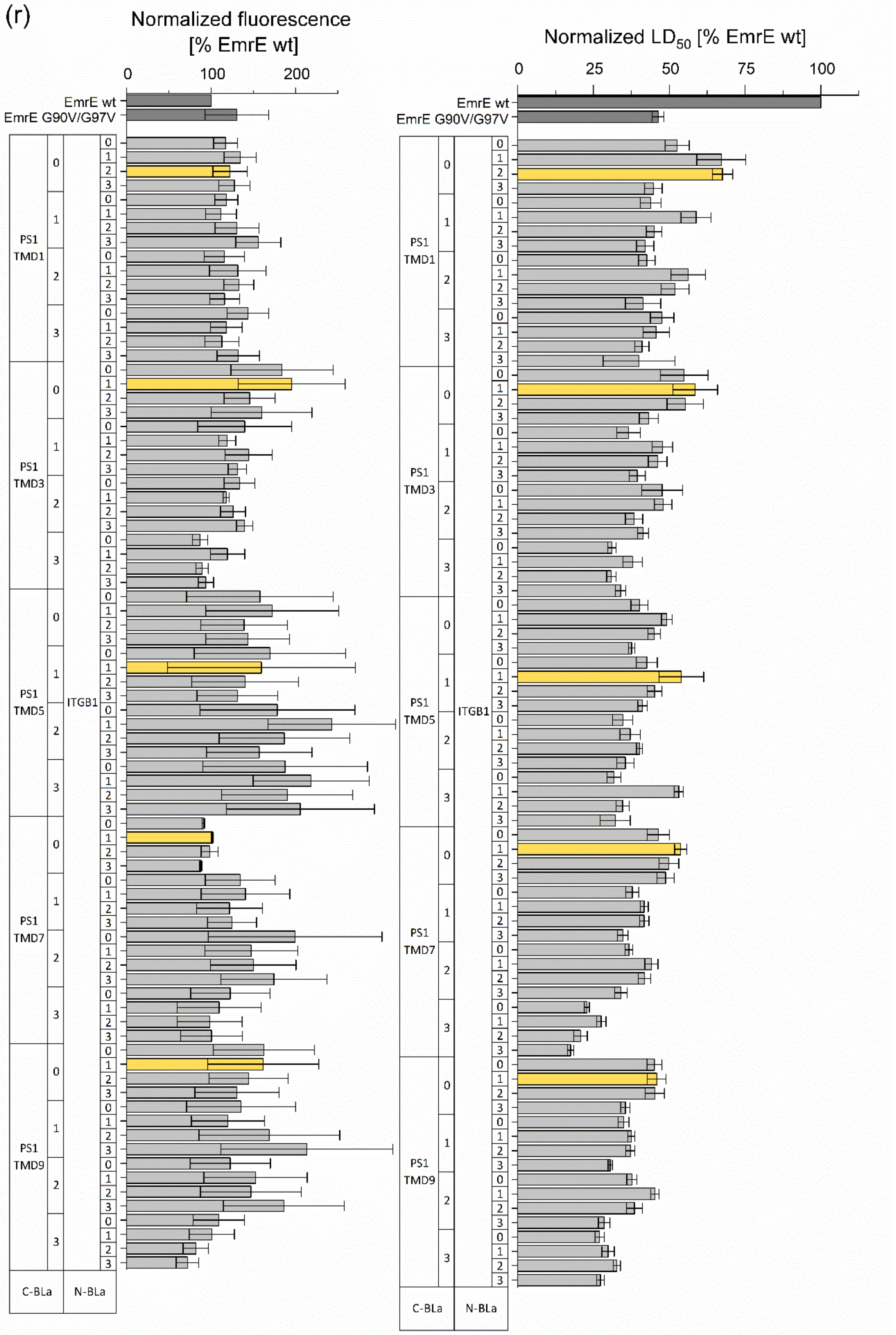

(r)
Normalized fluorescence [% EmrE wt]
0
100
200
EmrE wt
EmrE G90V/G97V
PS1 TMD1
PS1 TMD3
PS1 TMD5
PS1 TMD7
PS1 TMD9
ITGB1
C-BLa
N-BLa
Normalized LD50 [% EmrE wt]
0
25
50
75
100

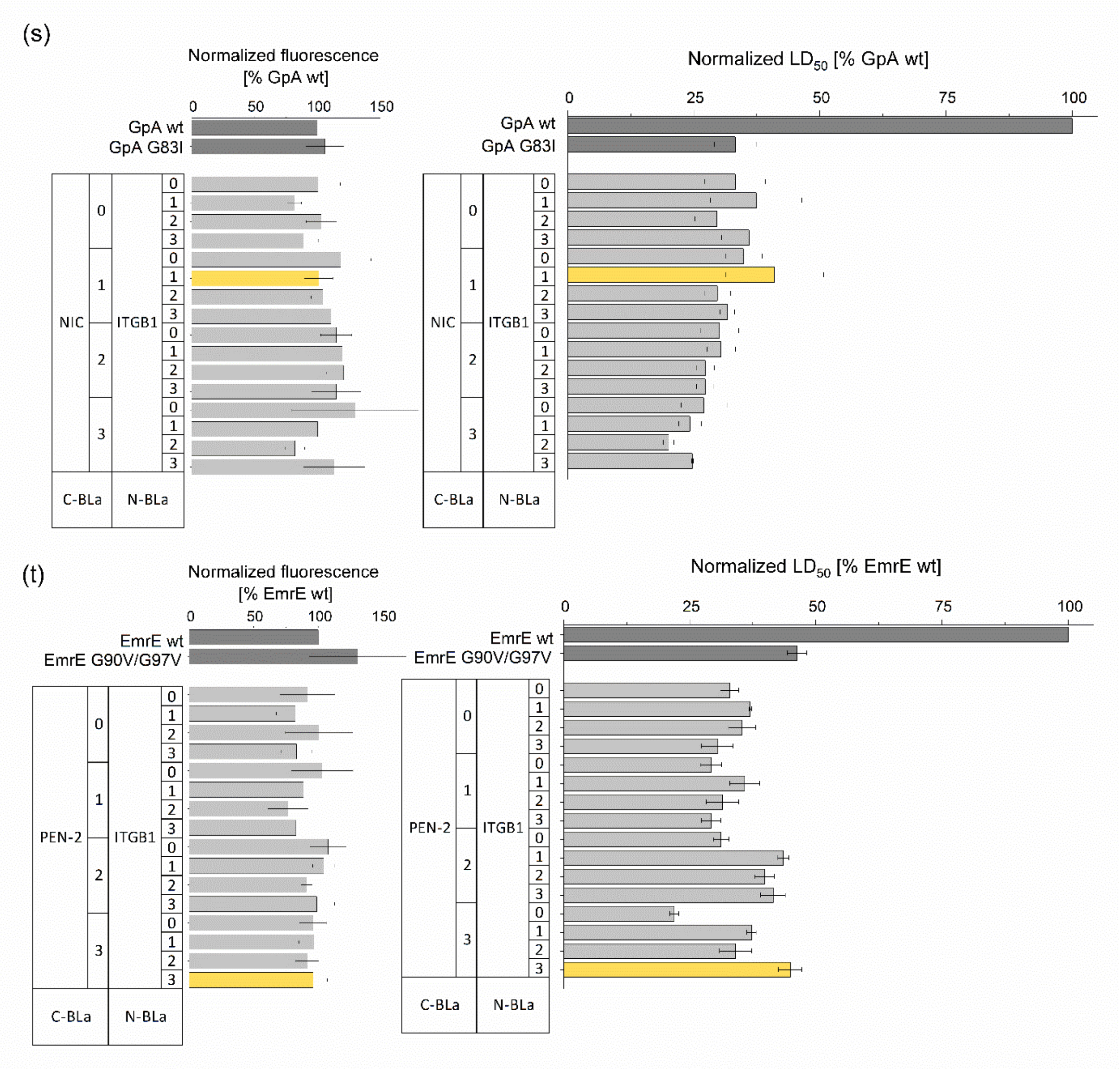

Figure S2. Heterotypic interactions of all investigated pairings of non-/substrate TMDs and γ-secretase TMDs. The strongest interaction in any given pairing is depicted in color and reproduced in Fig. 3 and Fig. 4. The right or upper panels show the LD_{50} values of the tested pairs while the left or lower panels represent the respective GFP expression controls. Parts **(a)** – **(d)** show all pairs of the APP TMD and the TMDs of PS1, nicastrin, and PEN-2. Parts **(e)** – **(h)** show all pairs of the Notch 1 TMD and the TMDs of PS1, nicastrin, and PEN-2. Parts **(i)** – **(l)** show all pairs of the N-cadherin TMD and the TMDs of PS1, nicastrin, and PEN-2. Parts **(m)** – **(p)** show all pairs of the ErbB4 TMD and the TMDs of PS1, nicastrin, and PEN-2. Parts **(q)** – **(t)** show all pairs of the integrin β1 TMD and the TMDs of PS1, nicastrin, and PEN-2. Means ± SEM, n = 3-6. The color code corresponds to that Figs. 3 and 4.

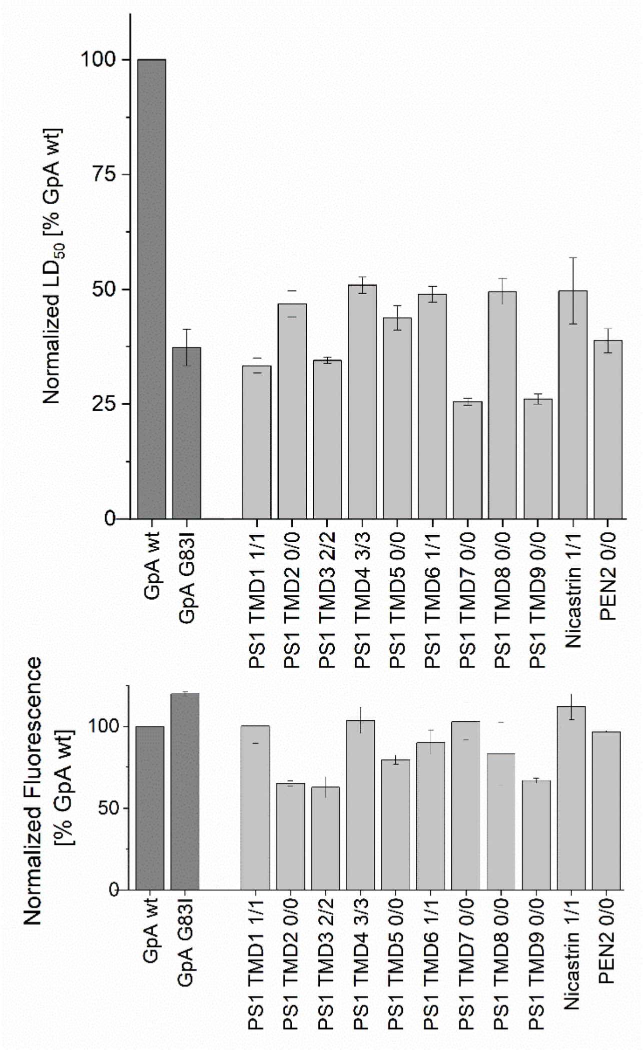

Figure S3. Homodimerization of γ-secretase TMDs. Upper panel: Strength of homotopic interactions of γ-secretase TMDs normalized to the homodimerization signal of GpA, used as reference. Lower panel: GFP expression controls of the respective pairs normalized to the GFP expression of GpA. Note that the frames tested here correspond to those frames yielding the highest heteromerization signals given in Fig. 3. Means ± SEM, n = 3.

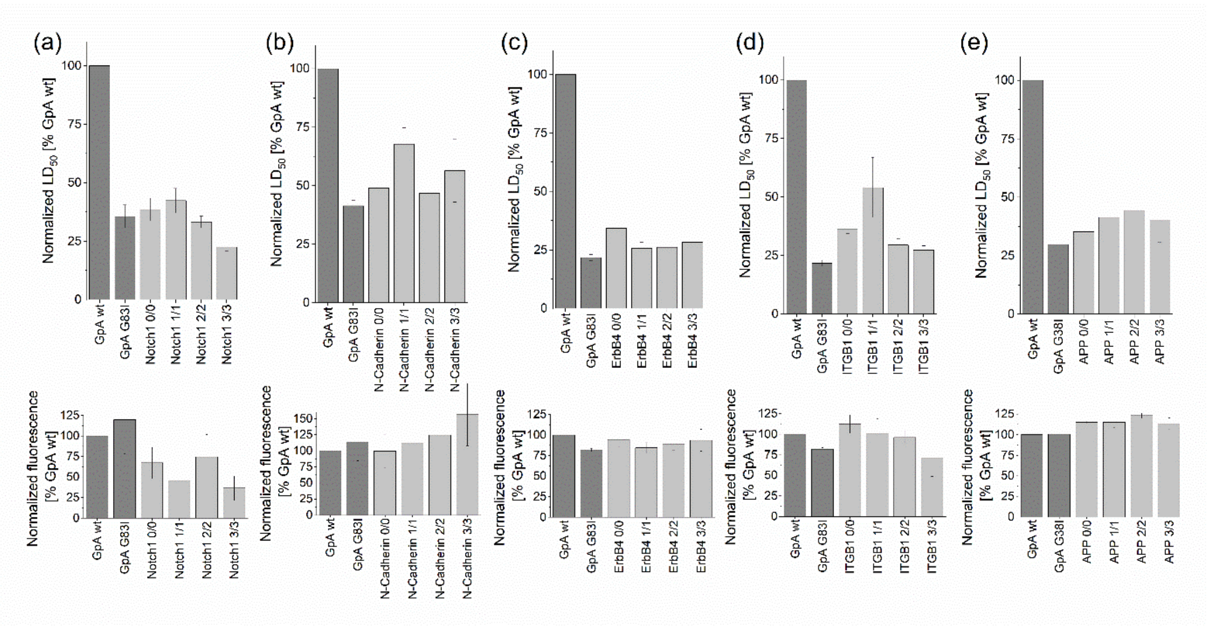

Figure S4. Homodimerization of (non)-substrate TMDs. Upper panels: strength of homotopic interaction of the Notch 1 **(a)**, N-cadherin **(b)**, ErbB4 **(c)**, integrin β1 **(d)**, or APP **(e)** TMD. Lower panels: respective GFP expression controls. All values are normalized to the homodimerization signal or GFP expression of the GpA TMD, respectively. Means ± SEM, n = 3-4.

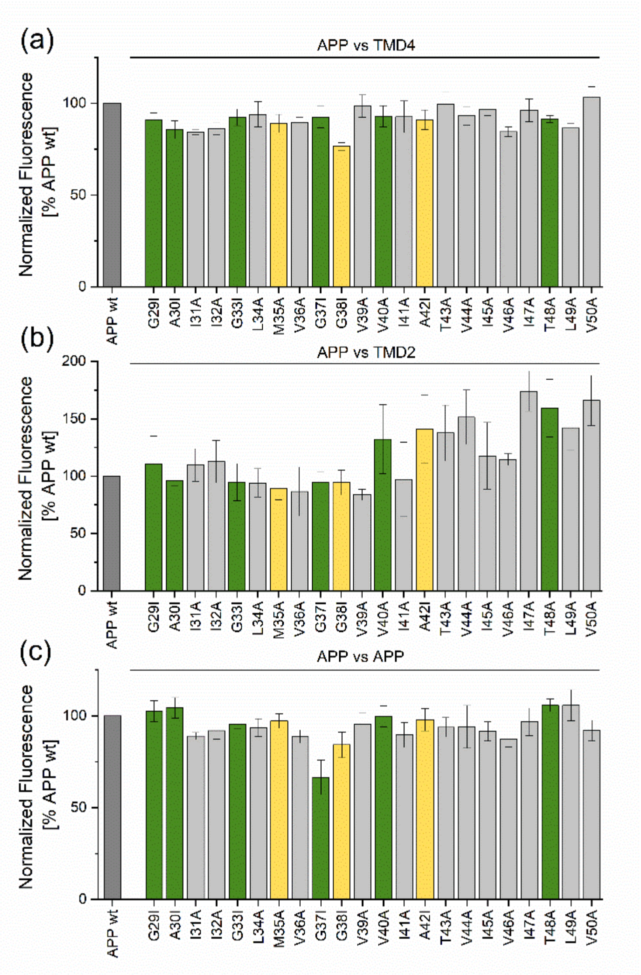

Figure S5. GFP expression controls corresponding to the mutational analysis of the heterotypic or homotypic interactions of the APP TMD. **(a)** GFP expression controls of APP TMD (frame 2) mutants paired with the PS1 TMD4 (frame 3). **(b)** GFP expression controls of APP TMD (frame 2) mutants paired with the PS1 $^{125\text{-}146}$TMD2 (frame 0). **(c)** GFP expression controls of the homodimerization of APP TMD (frame 2) mutants normalized to the signal of wt. Values are normalized to the signal of APP wt in the respective part. Means ± SEM, n = 4-5. The corresponding LD_{50} values are shown in Fig. 5 using the same color coding.

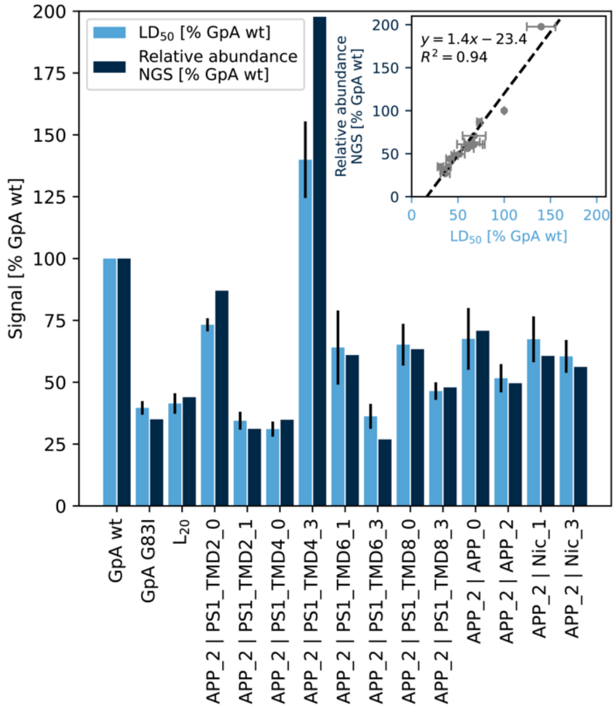

Figure S6. Validation of the library-based BLaTM system. The abundances of pairs of various reference TMD-TMD interactions, as determined by deep sequencing (black bars), are compared to manually measured LD_{50} values (blue bars). All values were normalized to the respective signals obtained with GpA wt. Inset: correlation analysis of both sets of data.

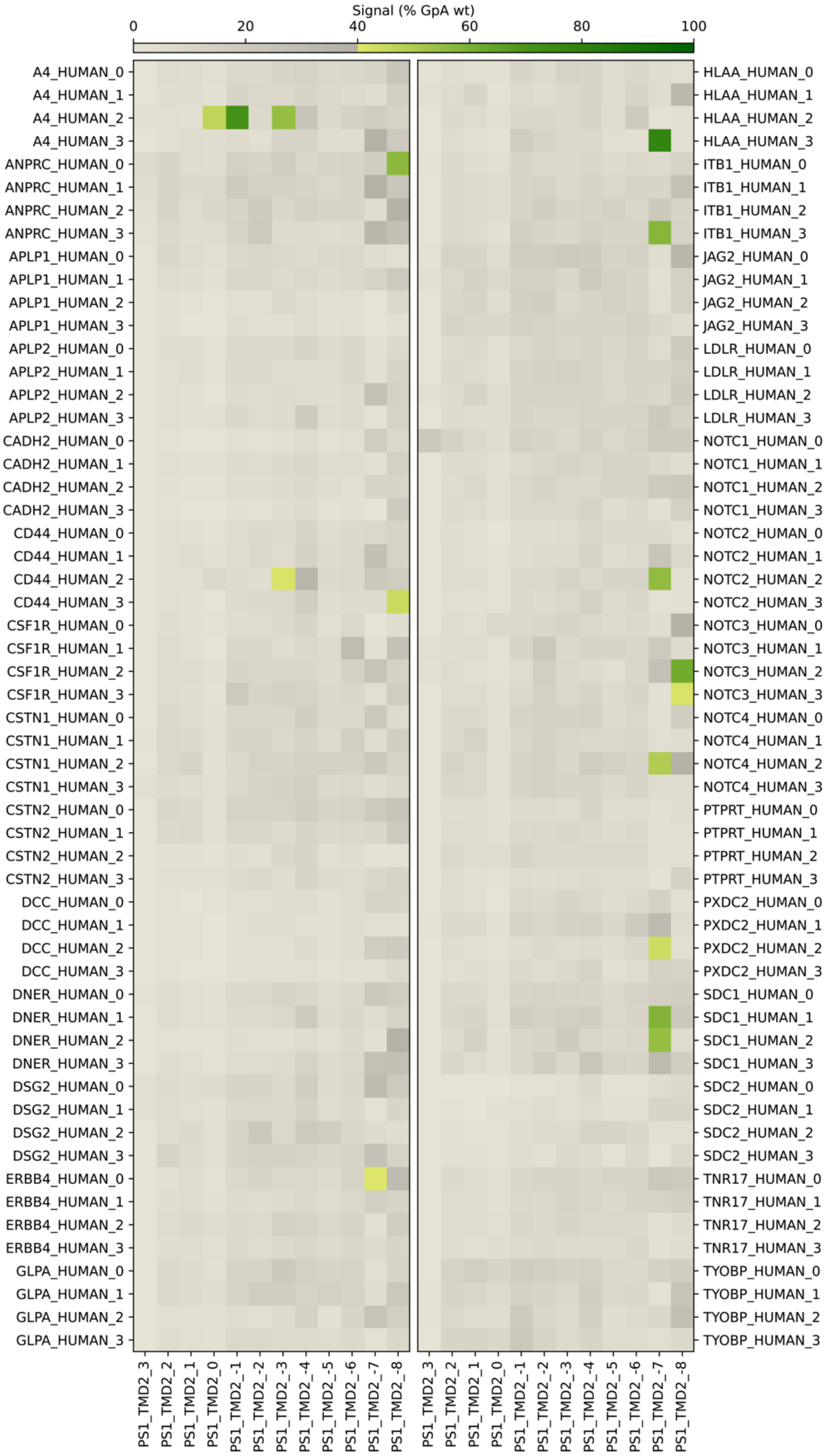

Figure S7. TMD-TMD interactions of various parts of the PS1 TMD2 with substrate TMDs, as examined with the BLaTM library screening system. The heatmap depicts the abundances of pairs obtained with all tested combinations of sequences and frames. Abundances in excess of 40% of the abundance seen with the GpA positive control are depicted in green and were confirmed by manual testing as shown in Fig. 6.

Supplemental reference

1. Bai, X. C.; Yan, C.; Yang, G.; Lu, P.; Ma, D.; Sun, L.; Zhou, R.; Scheres, S. H.; Shi, Y., An atomic structure of human gamma-secretase. *Nature* **2015,** 525, (7568), 212-7.
